# Identifying cell-to-cell variability in internalization using flow cytometry

**DOI:** 10.1098/rsif.2022.0019

**Published:** 2022-05-25

**Authors:** Alexander P. Browning, Niloufar Ansari, Christopher Drovandi, Angus P. R. Johnston, Matthew J. Simpson, Adrianne L. Jenner

**Affiliations:** ^1^ School of Mathematical Sciences, Queensland University of Technology, Brisbane, Australia; ^2^ ARC Centre of Excellence for Mathematical and Statistical Frontiers, Queensland University of Technology, Brisbane, Australia; ^3^ QUT Centre for Data Science, Queensland University of Technology, Brisbane, Australia; ^4^ Drug Delivery, Disposition and Dynamics, Monash Institute of Pharmaceutical Sciences, Monash University, 399 Royal Parade, Parkville, Victoria 3052, Australia

**Keywords:** heterogeneity, flow cytometry, internalization, endocytosis, noise, approximate Bayesian computation

## Abstract

Biological heterogeneity is a primary contributor to the variation observed in experiments that probe dynamical processes, such as the internalization of material by cells. Given that internalization is a critical process by which many therapeutics and viruses reach their intracellular site of action, quantifying cell-to-cell variability in internalization is of high biological interest. Yet, it is common for studies of internalization to neglect cell-to-cell variability. We develop a simple mathematical model of internalization that captures the dynamical behaviour, cell-to-cell variation, and extrinsic noise introduced by flow cytometry. We calibrate our model through a novel distribution-matching approximate Bayesian computation algorithm to flow cytometry data of internalization of anti-transferrin receptor antibody in a human B-cell lymphoblastoid cell line. This approach provides information relating to the region of the parameter space, and consequentially the nature of cell-to-cell variability, that produces model realizations consistent with the experimental data. Given that our approach is agnostic to sample size and signal-to-noise ratio, our modelling framework is broadly applicable to identify biological variability in single-cell data from internalization assays and similar experiments that probe cellular dynamical processes.

## 1. Introduction

Endocytosis is the primary means by which cells uptake, or internalize, drugs, viruses and nanoparticles [1–5]. Single-cell *in vitro* analysis of internalization and similar dynamical processes reveals significant cell-to-cell variability in otherwise homogeneous cell populations [6–12]. Such heterogeneity is ubiquitous to biology and essential to life, allowing for robust decision-making, development and adaptation of cell populations to environmental uncertainty [13–17]. From a clinical perspective, heterogeneity in drug uptake and response is considered a leading contributor to treatment variability and resistance [18–20]. The challenges of working with data that comprise instrument noise and background fluorescence which often obfuscate biological variability means that it is relatively common for quantitative analysis of internalization to neglect heterogeneity [21,22]. Exacerbating these issues is a corresponding lack of mathematical tools that account for cell-to-cell variability and measurement noise while also providing information about the uncertainty in inferences and predictions drawn from noisy data.

**Figure 1.**
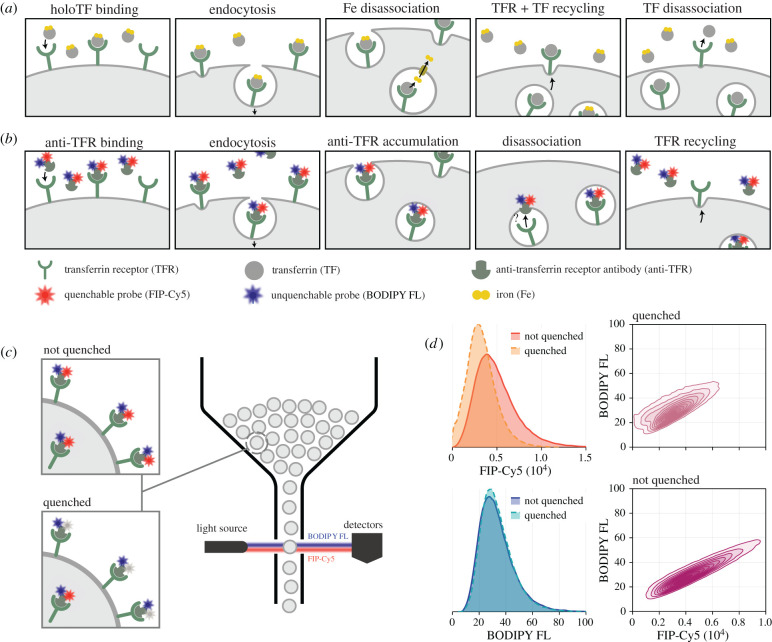
Internalization dynamics and corresponding experimental assay. (*a*) Internalization of transferrin, a protein responsible for the uptake of iron by cells. Iron-saturated transferrin (holoTF) binds to receptors on the cell surface and is internalized through *endocytosis*. In the low pH of endosomes, iron disassociates before the transferrin–receptor complex recycles to the cell surface and iron-free transferrin disassociates. (*b*) A corresponding internalization assay. Anti-transferrin receptor antibody (anti-TFR) dual-labelled with BODIPY FL and fluorescent internalization probe (FIP)-Cy5 replaces iron-loaded transferrin and is internalized through clathrin-mediated endocytosis. Experimental observations suggest that a small proportion of labelled antibody disassociates inside the cell, allowing receptor recycling and the accumulation of antibody inside the cell. (*c*) A quencher dye switches off fluorescence of surface-bound FIP-Cy5, providing information relating to the proportion of antibody that has internalized. Single-cell measurements of fluorescence from both probes are collected using flow cytometry. (*d*) Flow cytometry data obtained *t* = 10 min after antibody are introduced. Since variability in the data is predominantly biological, data from each fluorescent label are highly correlated. Univariate distributions shown are normalized (i.e. integrate to unity), and comparisons for all experimental time points are provided in electronic supplementary material, S1.

Modern analysis technologies, including flow cytometry, allow the high-throughput collection of data from experiments that probe internalization at rates exceeding a thousand cells per second (figure 1) [23]. In an internalization assay, material labelled with fluorescent probes is incubated with cells and internalized through pathways responsible for the uptake of material by cells, such as through clathrin-mediated endocytosis (figure 1*a*,*b*) [24,25]. The fluorescence of surface-bound probes can be switched off by introducing a quencher dye, or the fluorescence of internalized probes altered due to the lower pH in early endosomes [21,24], providing quantitative information relating to the amount of material internalized. Flow cytometry provides measurements related to the total and internalized amount of material at various time points (figure 1*c*,*d*). In contrast to methods that capture single-cell time-lapse data using microscopy [7,26], flow cytometry provides single-cell snapshot data, sacrificing information relating to individual trajectories for significantly higher sample sizes (often of the order of several million cells). While previous studies have shown that measurement noise introduced by the flow cytometry electronics and background autofluorescence are not insignificant, variability in the data is primarily biological [11,27–31]. We confirm this by performing an internalization assay with a dual-labelled fluorescent probe, finding that measurements are highly correlated, indicating a shared source of variability (figure 1*d*).

Mathematical and statistical techniques allow quantitative analysis of transient dynamics, heterogeneity and measurement noise. As the number of molecules internalized by each cell is relatively large, single-cell trajectories describing the relative amount of material internalized can be accurately described by deterministic models derived through kinetic rate equations. Ordinary differential equation (ODE) constrained Bayesian hierarchical and random effects models incorporate cell-to-cell variability through a parameter hierarchy where distributions parametrized by hyperparameters describe cell-level properties [32–34]. Both individual cell properties and hyperparameters are estimated during calibration of hierarchical models to data, presenting a significant computational challenge for the large sample sizes provided by flow cytometry data. In the mathematical literature, so-called heterogeneous [35] or random [36] ODEs and populations of models [37] make similar assumptions, often without assuming a parametric distribution of cell properties [9,38,39]. Issues presented by large sample sizes can be avoided by calibrating models using the empirical distribution of the data (through, for example, kernel density estimates) [35], an approach that provides point estimates but neglects inferential uncertainty and poses a challenge when the signal-to-noise ratio in the data is not sufficiently high.

In this study, we develop a mathematical model of internalization that captures cell-to-cell variability by describing cell properties—specifically, the number of receptors, the internalization rate and the recycling rate of each cell—as jointly distributed random variables. To describe non-biological sources of variability from flow cytometry measurements of an internalization assay, we couple the dynamical model to a probabilistic observation process that captures autofluorescence and measurement noise. We take a Bayesian approach to parameter estimation and develop a novel approximate Bayesian computation (ABC) [40–42] algorithm that matches distributional information from flow cytometry measurements, with the goal of identifying sources of cell-to-cell variability that are consistent with experimental observations. Given that ABC relies only on model realizations and not the structure of the model itself, this approach is agnostic to the signal-to-noise ratio, the complexity of the probabilistic observation process, as well as the sample size. Furthermore, ABC allows us to obtain both point parameter estimates and information relating to inferential uncertainty, which provides information about the range of parameters that produce model realizations consistent with the experimental observations.

We demonstrate our approach by studying heterogeneity in the internalization of anti-transferrin receptor (anti-TFR) antibody in C1R cells, a human B lymphoblastoid line. Data comprise potentially noisy flow cytometry measurements from an internalization assay developed in our previous work, specific hybridization internalization probe (SHIP) (figure 1*b*,*c*) [21,43]. Measurements are collected from anti-TFR antibody dual labelled with BODIPY FL and fluorescent internalization probe (FIP)-Cy5. We take measurements both with and without a quencher dye, which switches off the fluorescence of surface-bound FIP-Cy5 without affecting internalized FIP-Cy5 or the BODIPY FL signal (results in figure 1*d* show only very minor experimental variability in BODIPY FL between samples that are quenched and not). Therefore, we obtain jointly distributed data that comprise noisy measurements of the total and internalized amount of antibody in each cell (figure 1*c*,*d*). Snapshots are collected from samples that are incubated with antibody-saturated medium for various periods of time to provide measurements relating to both the total and internalized amounts of antibody present on each cell. Using our mathematical model, we are able to identify key sources of biological variability and provide predictions that give insight into how the uptake of material varies between cells. Importantly, our approach to parameter inference enables us to quantify the uncertainty in inferences made, allowing us to provide experimental design guidance.

## 2. Results

### 2.1. Dynamical model of internalization

**Figure 2.**
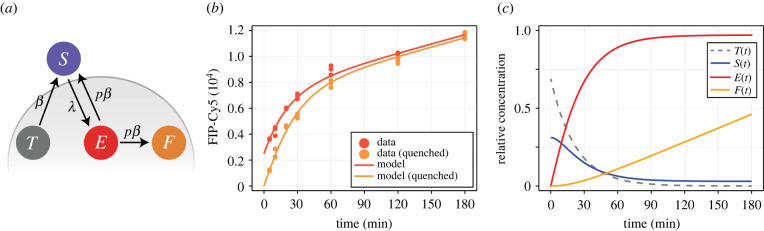
Dynamical model of internalization and recycling matches experimental data. (*a*) The dynamical model describes the relative concentration of internal, transferrin-bound receptors, *T* (grey); surface antibody-bound receptors, *S* (blue); internal antibody-bound receptors, *E* (red); and internal free antibody, *F* (orange). (*b*) Geometric mean of FIP-Cy5 fluorescence measurements for samples that are not quenched (red) and those that are (orange) at various time points. The dynamical model is calibrated using maximum-likelihood estimation, with the solution shown (solid curve). (*c*) Solution to the mathematical model at the maximum-likelihood estimate (table 1).

We describe the internalization of antibody and the recycling of receptors using a compartment model. Given that the concentration of antibody in the surrounding medium is sufficiently high, we assume that the association rate of antibody to free receptors on the cell surface is much higher than the kinetic rates of internalization and recycling (electronic supplementary material, S3). Therefore, we describe the number of antibody–receptor complexes on the cell surface, *S*, and that endocytosed, *E*. Before incubation in antibody-saturated medium, endocytosed receptors are bound to transferrin. To capture this, we describe a pool of internal, transferrin-bound receptors, of size *T*. Experimental results (figure 2*b*) do not show the antibody concentration reaching a limiting concentration. This suggests at an accumulation of free antibody inside cells, of number *F*, with receptor recycling driving the continued uptake of antibody throughout the experiment. As the recycling kinetics of antibody-bound receptors are unknown, we assume that with small probability, *p*, endocytosed antibody-bound receptors recycle and antibody disassociates. These assumptions give rise to the dynamical model (figure 2*a*)

2.1 $$\begin{array}{cc} & T\overset{\beta }{\to }S\\ \mathrm{and}\;\;\;\; & S\overset{\lambda }{\to }E\overset{\;p\beta }{\to }S+F, \end{array}\}$$

where *β* (min^−1^) is the recycling rate and *λ* (min^−1^) is the internalization rate. It is also possible that endocytosed antibody, *E*, can return to the cell surface without disassociation from the receptor. However, we have not included this in our model as a recycled antibody–receptor complex is indistinguishable from that bound on the cell surface, *S*.

Given that the number of receptors in each cell is relatively large, equation (2.1) can be formulated as a linear ODE with exact solution **x**(*t*) = **x**_0_ exp(**M***t*), where **M** is a matrix of coefficients and **x**(*t*) denotes the number of molecules in each compartment (electronic supplementary material, S2). Initially, the system is in equilibrium, so

2.2 $$S(0)=\frac{\beta }{\lambda +\beta },$$

where molecule counts are taken with respect to the total number of receptors on the cell, denoted *R*, so *S*(0) + *T*(0) = 1.

### 2.2. Inference using mean fluorescence intensity measurements

Flow cytometry measurements are typically summarized using the geometric mean of the fluorescence intensity distribution, called the *geometric mean fluorescence intensity* (GMFI) (figure 2*b*). Cy5 GMFI measurements from samples that are not quenched are related to the total amount of antibody in the sample, *A*(*t*) = *S*(*t*) + *E*(*t*) + *F*(*t*), and measurements from quenched samples are related to the amount of internal antibody in the sample, *I*(*t*) = *E*(*t*) + *F*(*t*). In practice, quenching is imperfect, and a small proportion of surface-bound antibody retains fluorescence. We pre-estimate this *quenching efficiency*, *η*, by comparing the fluorescence intensity of quenched and not quenched samples of cells kept at 4°C, which inhibits internalization, finding that *η* ≈ 0.94 (electronic supplementary material, S4). Therefore, GMFI measurements can be modelled by

2.3 $$\begin{array}{rl} Q_{\mathrm{GMFI}}(t) & =\alpha_{1}A(t)+E_{Q}+\kappa_{1}\\ \;\mathrm{and}\;{\bar{Q}}_{\mathrm{GMFI}}(t) & =\alpha_{1}[I(t)+\eta S(t)]+E_{Q}+\kappa_{2}. \end{array}\}$$

Here, we denote by *Q*_GMFI_(*t*) GMFI measurements from the FIP-Cy5 (i.e. quenchable) probe in the samples that are not quenched, by ${\bar{Q}}_{\mathrm{GMFI}}(t)$ that of quenched samples and by *E*_*Q*_ the average autofluorescence. We capture variability in GMFI measurements, which are statistics of the full fluorescence distribution, by assuming measurement error *κ*_1_, *κ*_2_ ∼ Normal(0, *σ*^2^). The parameter *α*_1_ relates the antibody concentration to the fluorescence intensity measurement: *A*(*t*) = 1 corresponds to a GMFI measurement of *α*_1_ units. We refer to equation (2.3) as the *homogeneous model* since the dynamical parameters *λ* and *β*, and the number of receptors, *R*, do not vary cell-to-cell and are fixed for the population.

To assess the suitability of the dynamical model and provide a baseline to assess our model that captures biological heterogeneity, we calibrate equation (2.3) to experimental data using maximum-likelihood estimation. We tabulate estimates and confidence intervals approximated using the observed Fisher information in table 1, and show the model best fit in figure 2*b*.

**Table 1.** Parameter estimates and approximate confidence intervals for the homogeneous model. Approximate confidence intervals are calculated using the observed Fisher information matrix, calculated from the Hessian of the log-likelihood function [44].

parameter	estimate	95% CI	units
*λ*	0.106	(0.097, 0.116)	min^−1^
*β*	0.047	(0.043, 0.051)	min^−1^
*p*	0.068	(0.063, 0.072)	—
*α*_1_	7840	(7540, 8140)	fluorescence units

The homogeneous model provides a fit that qualitatively matches GMFI measurements from the experimental data (figure 2*b*), and all parameters are identifiable within relatively precise intervals (table 1). Estimates for the internalization and recycling rates suggest that a proportion of approximately

2.4 $$S(0)=\frac{\beta }{\lambda +\beta }\approx 0.31\;$$

of transferrin receptors lie on the surface at equilibrium. Estimates for *p* suggest that 6.8% (95% CI (6.3%, 7.2%)) of internalized antibody disassociates, allowing receptor recycling. This is also evident from simple observations of the experimental data, since the fluorescence intensity increases throughout the experiment, suggesting that a small proportion of receptors remain on the surface while antibody accumulates inside the cell (figure 2*c*).

### 2.3. Incorporating biological variability into dynamical model of internalization

We assume biological variability arises through both physical and physiological differences between cells in the population. Specifically, we allow number of receptors, *R*, and dynamical parameters *λ* and *β* to vary cell-to-cell. Without loss of generality, we set E(R)=1 so receptor and antibody counts are taken with respect to the average receptor count in the population. Given that *p* relates to a strictly chemical process governing the association of receptor to antibody, we assume that it does not vary cell-to-cell.

The properties of the *i*th cell are modelled by the random variable ***ξ***_*i*_ = (*R*_*i*_, *λ*_*i*_, *β*_*i*_). Given that we see no evidence of a subpopulation structure in the experimental data, we make the basic assumption that ***ξ****_i_* is unimodal. We expand on typical hierarchical modelling restrictions [34] by allowing cell properties *λ*_*i*_ and *η*_*i*_ to vary according to both normal and non-normal distributions. To do this, we describe *λ*_*i*_ and *η*_*i*_ as shifted Gamma variables parametrized in terms of their respective means, $\left(\mu_{\lambda },\mu_{\beta }\right)$, standard deviations, $\left(\sigma_{\lambda },\sigma_{\beta }\right)$, and skewnesses, $\left(\omega_{\lambda },\omega_{\beta }\right)$ (electronic supplementary material, S6). This approach allows us to recover normal distributions in the limit *ω* → 0 in addition to distributions with positive (*ω* > 0) and negative (*ω* < 0) skewnesses, and we note that it is relatively common to use Gamma distributions in their own right to describe heterogeneity in rate constants in biology [45,46]. The number of receptors, *R*_*i*_, is assumed to be shifted log-normally distributed [47]. To ensure positivity, we truncate ***ξ***_*i*_ so that *R*_*i*_, *λ*_*i*_, *β*_*i*_ ≥ 0. The untruncated marginal distributions are given by

2.5 $$\begin{array}{cc} & R_{i}∼\mathrm{ShiftedLogNormal}(\mu_{R},\sigma_{R}),\\ & \lambda_{i}∼\mathrm{ShiftedGamma}(\mu_{\lambda },\sigma_{\lambda },\omega_{\lambda })\\ \;\mathrm{and}\; & \beta_{i}∼\mathrm{ShiftedGamma}(\mu_{\beta },\sigma_{\beta },\omega_{\beta }). \end{array}\}$$

We model the dependence structure of ***ξ***_*i*_ with a Gaussian copula parametrized by the correlation matrix

2.6 $$P=\left(\begin{array}{ccc} 1 & \rho_{R\lambda } & \rho_{R\beta }\\ \rho_{R\lambda } & 1 & \rho_{\lambda \beta }\\ \rho_{R\beta } & \rho_{\lambda \beta } & 1 \end{array}\right).$$

To ensure **P** remains positive definite, we infer *ρ*_*Rλ*_, *ρ*_*λβ*_ and ${\tilde{\rho }}_{R\beta }$ (all constrained to the interval (−1, 1)) where

2.7 $$\rho_{R\beta }=\rho_{R\lambda }\rho_{\lambda \beta }+{\tilde{\rho }}_{R\beta }\sqrt{\left(1-\rho_{R\lambda }^{2})(1-\rho_{\lambda \beta }^{2}\right)}.$$

Therefore, *ρ*_*Rλ*_ (and similarly for *ρ*_*Rβ*_ and *ρ*_*βλ*_) describes the strength of the correlation between the number of receptors, *R*, and internalization rate, *λ*. We note that this model of dependence would be equivalent to the standard approach to model ***ξ***_*i*_ as a multivariate normal [34] with correlation matrix **P** if the marginals where also normally distributed. In electronic supplementary material, S7, we provide full details of how samples of ***ξ*** are obtained.

The *heterogeneous model* is a random ODE model where **x**(*t*) and its constituents are random variables [36]. For example, *A*(*t*) is a random variable describing the distribution of bound-antibody present on a cell at time *t*.

### 2.4. Statistical model for flow cytometry data

Measurement noise in flow cytometry is primarily attributable to shot noise introduced from the photomultiplier tubes (PMT noise) that convert the photon signal to an amplified, analogue electrical signal. Recent studies suggest that the square coefficient of variation of such noise is approximately constant [30], so we model shot noise with uncorrelated white noise (i.e. Gaussian), with variance proportional to the true signal. The second source of noise is cellular autofluorescence, where the laser used to excite the labelled antibody can excite other molecules in the cell, leading to a background autofluorescence where signal is present in the absence of antibody. We build an empirical distribution of autofluorescence (*E*_*Q*_, *E*_*U*_) using a sample where cells have not been introduced to labelled antibody (electronic supplementary material, S5).

We denote measurements from the FIP-Cy5 probe, which is quenchable, by *Q*(*t*), and the BODIPY FL probe, which is not quenchable, by *U*(*t*). Therefore,

2.8 $$Q(t)=\alpha_{1}A(t)R+\varepsilon_{1}\sqrt{\alpha_{1}A(t)R}+E_{Q}$$

and

2.9 $$U(t)=\underset{\text{Antibody}}{\underbrace{\alpha_{2}A(t)R}}+\underset{\text{PMT noise}}{\underbrace{\varepsilon_{2}\sqrt{\alpha_{2}A(t)R}}}+\underset{\text{Autofluorescence}}{\underbrace{E_{U},}}$$

where $\varepsilon_{1},\varepsilon_{2}∼\mathrm{Normal}(0,\sigma_{k}^{2})$. Similarly, the measurements from quenched samples are given by

2.10 $$\begin{array}{rl} \bar{Q}(t) & =\alpha_{1}[I(t)+(1-\eta )S(t)]R\\ & \;+\varepsilon_{1}\sqrt{\alpha_{1}[I(t)+(1-\eta )S(t)]R}+E_{Q} \end{array}$$

and

2.11 $$\bar{U}(t)=\alpha_{2}A(t)R+\varepsilon_{2}\sqrt{\alpha_{2}A(t)R}+E_{U}.$$

### 2.5. Calibration and uncertainty quantification

We take a Bayesian approach to parameter estimation calibrating the noisy heterogeneous model to SHIP assay data using a novel ABC [40–42] algorithm that matches the empirical distribution of flow cytometry measurements, under the assumption that measurements from each probe are linearly correlated. There are two primary factors that motivate our preference for this approach. First, the approach is agnostic to the sample size as we work with the observed empirical distributions directly, rather than individual samples. Second, we are not limited in the complexity of the statistical model and can, therefore, work with a statistical model of measurement noise motivated by the actual electronics in the collection method, in addition to empirical, and not approximate, distributions of autofluorescence.

Given a set of experimental observations X, we encode knowledge about the model parameters ***θ*** in the *posterior distribution*, given by

2.12 $$\underset{\text{posterior}}{\underbrace{\;p(\theta |X)}}\propto \underset{\text{likelihood}}{\underbrace{\;p(X|\theta )}}\underset{\text{prior}}{\underbrace{\;p(\theta )}}.$$

**Figure 3.**
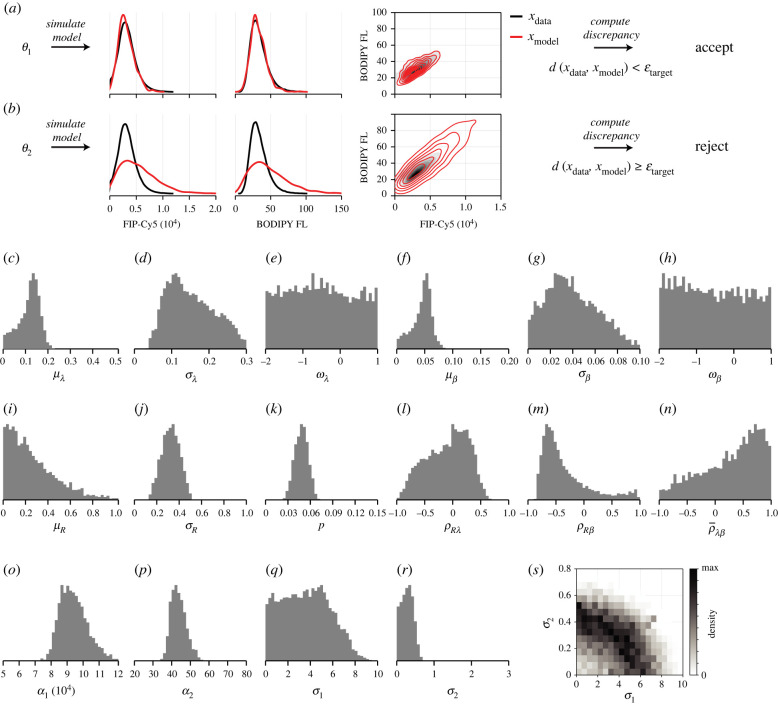
Model calibration and uncertainty quantification using ABC MCMC. (*a*,*b*) In ABC, data are compared to model simulations using a weighted sum of Anderson–Darling distances and discrepancy in the correlations. (*a*) Parameter combinations that produce model realizations sufficiently similar to the experimental data, i.e. ***θ***_1_, are accepted as posterior samples. (*b*) Parameter combinations that do not, i.e. ***θ***_2_, are rejected. (*c*–*s*) *Posterior samples* obtained using ABC MCMC represent parameter combinations that produce realizations of the model that are similar to experimental observations. Chains are initiated at the global minimum identified using ABC SMC, and every 100th sample is retained. All axis limits for univariate distributions correspond to the prior support (uniform priors are used). Parameter descriptions and MCMC diagnostics are given in electronic supplementary material, S9.

Here, *p*(***θ***) denotes the *prior distribution*, which encodes prior parameter knowledge. In our work, we take a standard approach and set the prior to be uniform with independent components [48] that correspond to the axis limits in figure 3. We choose parameter bounds to reflect either physical constraints on parameters (i.e. all correlations are bounded and rates, standard deviations, proportionality constants are positive) or realistic bounds (for example, we expect the distributions of *λ* and *β* to be negatively skewed so that support is low, but non-zero, at zero if internalization or recycling is inhibited in a small proportion of cells).

In ABC, we approximate the posterior distribution using the ABC posterior

2.13 p(θ|X)≈p(θ|d(X,Y(θ))<ε).

Here, Y(θ) denotes synthetically generated observations of the model using parameters ***θ***, *d*( · , · ) is a *discrepancy measure* that measures how close synthetically generated observations lie to the experimental data (figure 3*a*,*b*), and *ɛ* is a parameter that describes the maximum discrepancy at which synthetic observations are judged to be close. Our choice of discrepancy measure, *d*( · , · ), matches a weighted sum of discrepancies between univariate distributions (using the Anderson–Darling distance [49]) and discrepancies in the correlation of FIP-Cy5 to BODIPY FL signal. The weights in *d*( · , · ) are chosen so that the contribution from the univariate distribution and correlation discrepancies are similar in magnitude. Synthetic datasets are generated using *n* = 1000 cells per observation time, per condition (quenched or not quenched), and *ɛ* is chosen based on a pilot inference using a sequential Monte Carlo (SMC) algorithm [50]. Full details of the discrepancy measure and sampling algorithm are given in electronic supplementary material, S8.

**Figure 4.**
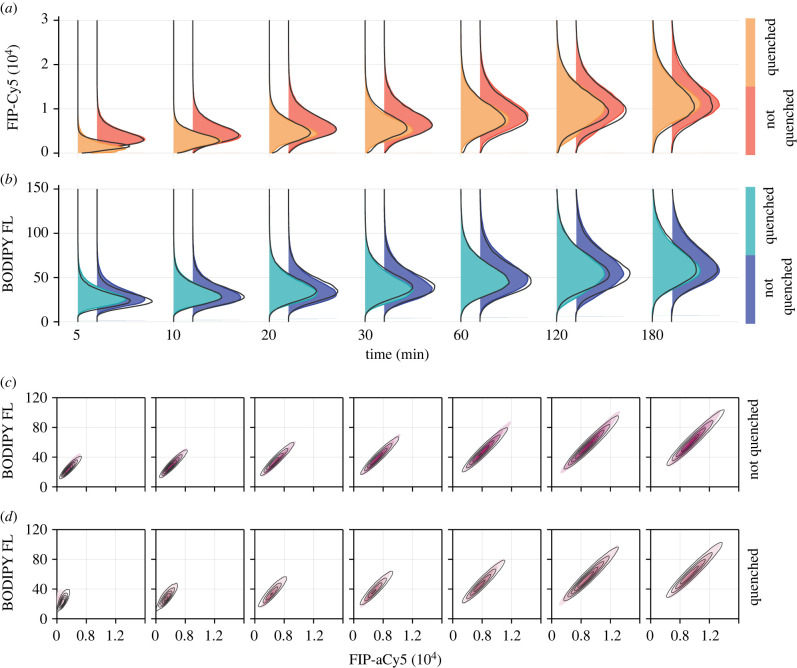
Mathematical model captures variability in experimental data. (*a*,*b*) Univariate kernel density estimate of the fluorescence intensity distribution from (*a*) the Cy5 probe, which is susceptible to the quencher dye, and (*b*) BODIPY FL, which is not. In each case, the distribution from the quenched experiment is shown to the left in the lighter colour. (*c*,*d*) Bivariate kernel density estimates of the joint fluorescence intensity distributions for FIP-Cy5 and BODIPY FL measurements. The model prediction from a synthetic dataset of 100 000 cells per observation time, per condition, produced using the model best fit is overlaid in black. Univariate distributions are normalized to the mode of the experimental measurements. In electronic supplementary material, S10, we show additional results comparing experimental measurements to model predictions from 100 randomly resampled parameter combinations from the posterior distribution.

In figure 3, we plot posterior samples from four independent tuned Markov chain Monte Carlo (MCMC) chains thinned to a total of 400 000 samples, providing an effective sample size of at least 1000 per parameter. To visualize model predictions, we compute a point estimate by further thinning the chains to a total of 400 samples, and identifying the parameter set that produces the lowest average discrepancy from 100 model realizations. Model predictions at the point estimate are shown alongside experimental data in figure 4. MCMC diagnostics, parameter descriptions and best-fit estimates are given in electronic supplementary material, S9.

### 2.6. Heterogenous model captures biological variability

The heterogeneous model produces realizations that agree with flow cytometry measurements, matching both marginal and jointly distributed data from both probes (figure 4). Minor discrepancies in univariate distributions highlight the main sources of unaccounted error; for example, error relating to the precise time at which internalization is ceased and error relating to flow cytometry gating.

Samples relating to the skewness of the internalization and recycling rate distributions, $\omega_{\lambda }$ and $\omega_{\beta }$, respectively (figure 3*c*,*f*), show that information in the experimental data is insufficient to identify the shape of the internalization and recycling rate distributions. While the precision to which we can identify the variance of each rate, $\sigma_{\lambda }$ and $\sigma_{\beta }$ (figure 3*b*,*e*), is limited, it is clear that $\sigma_{\lambda }>0.057$ (lower bound on a 95% CrI), providing evidence to suggest heterogeneity in the internalization rate. While *p* has the same interpretation between the heterogeneous and homogeneous models, the estimates from the heterogeneous model, p=4.7% (95% CrI (3.0%, 6.5%)), are lower with a greater amount of uncertainty than in the homogeneous model.

**Figure 5.**
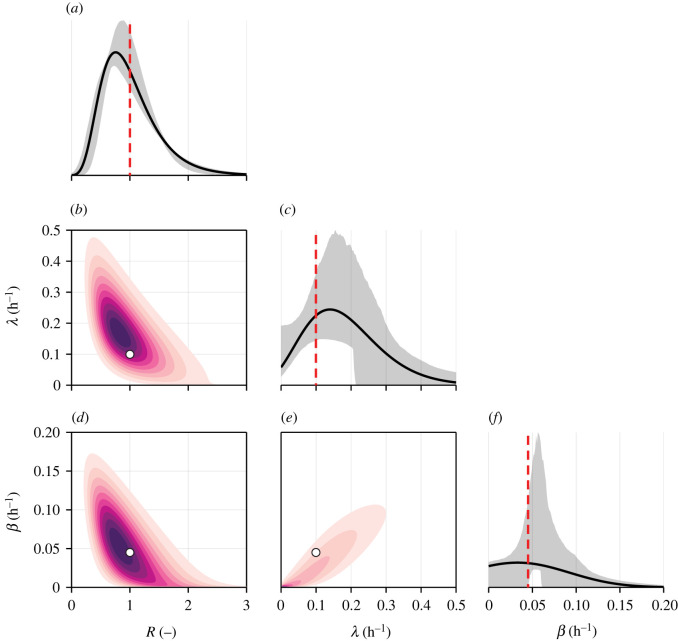
Inferred parameter distributions and associated uncertainty. Inferred distribution of (*a*) the relative number of receptors, *R*; (*c*) the internalization rate, *λ*; and (*f*) the recycling rate, *β*. Shown are the distributions at the best fit (black), a 95% credible interval of the respective probability density functions constructed from re-sampled MCMC samples (grey), and estimates from the homogeneous model (red). (*b*,*d*,*e*) Bivariate distributions at the best fit. Estimates from the homogeneous model are shown in white.

In figure 5, we plot the inferred distributions of *R*, *λ* and *β*; that is, distributions describing cell-to-cell variability that are able to model realizations consistent with the experimental data. To visualize uncertainty in estimates of these distributions, we show a 95% credible internal (CrI) for the univariate probability density functions by resampling from the posterior distribution. Compared to distributions of the dynamical parameters *λ* and *β*, the distribution of the relative receptor count, *R*, is identified with much greater precision (figure 5*a*). *R* does not feature in the dynamical model and is, therefore, less sensitive to issues relating to model misspecification. While results in figure 3*j*–*l* show relatively large uncertainty in the correlations between parameters, it appears likely that the receptor count and recycling rate are negatively correlated (83% of posterior samples have *ρ*_*Rβ*_ < 0). Parameters identified in the homogeneous model based on GMFI measurements are contained within high-density regions of the inferred distributions in the heterogeneous model. This is also the case when estimates are compared to bivariate distributions in figure 5*d*–*f*; however, the interpretation of the homogeneous model parameters in the context of data with significant heterogeneity is unclear, highlighting the importance of modelling biological variability when interpreting flow cytometry data of dynamical processes like internalization.

The data appear insufficient to distinguish between PMT noise from each fluorescent channel. Initial examination of estimates for the relative magnitude of the noise from the quenchable (FIP-Cy5) and unquenchable (BODIPY FL) probe signals in figure 3*o*–*p* suggests that the no-noise model may be appropriate, lending the study to analysis of models that assume negligible noise [35]. However, the joint distribution of *σ*_1_ and *σ*_2_ (figure 3*s*) reveals an elliptical region, suggesting that the model requires PMT noise in the signal from *at least one* probe. Similar phenomena are observed in error-in-variables or total-least-squares problems, where errors are introduced in both independent and dependent variables, and only the ratio of the error variances is identifiable [51].

### 2.7. Model predicts unobservable measurements

A primary goal of flow cytometry analysis is to quantify the amount of fluorescent material present in a sample. In the context of an internalization assay, we are interested in the *proportion* of material internalized through time. By accounting for variability introduced through receptor count, PMT noise and autofluorescence, we are able to better quantify the amount, or proportion internalized, of antibody compared with standard approaches.

Since it is not possible to collect noise-free data relating to the joint distribution of *I*(*t*), provided from quenched samples, and *A*(*t*), provided from sampled that are not quenched, statistics such as the proportion of antibody internalized by each cell cannot be directly measured. Rather, such statistics are typically estimated as

**Figure 6.**
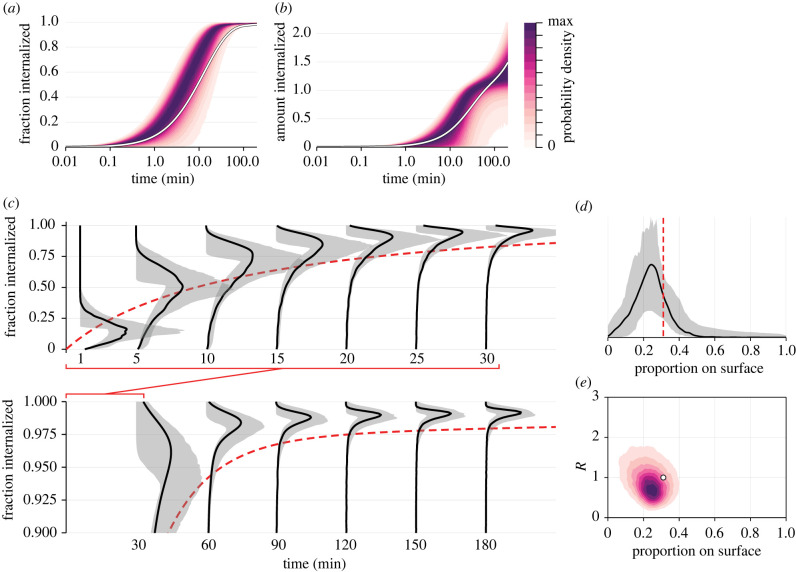
Model predicts unobservable measurements. Using the calibrated mathematical model, we can predict the time evolution of the distribution of (*a*,*c*) fraction of antibody inside the cell; (*b*) amount of antibody internalized relative to the average number of receptors on a cell, *I*(*t*); and (*d*,*e*) proportion of receptors on the cell surface at equilibrium. (*a*,*b*,*e*) The distribution at the model best fit, which is shown in black in all other plots; (*c*,*d*) additionally show 95% credible intervals constructed from MCMC samples. (*c*) To compare predictions with the homogeneous model, we show *A*(*t*)/*I*(*t*) predicted by the homogeneous model (red-dashed) using only GMFI measurements. In (*a*–*c*), distributions are normalized to the mode of the distribution at the model best fit.

2.14 $$I_{\mathrm{frac}}(t)=\frac{I_{\mathrm{GMFI}}(t)}{A_{\mathrm{GMFI}}(t)},$$

where *I*_GMFI_(*t*) and *A*_GMFI_(*t*) are scalar estimates of the average proportion of internal and total antibody estimated using GMFI [7]. Using our calibrated heterogeneous model, we can predict the *distribution* of material internalized through time by simulating the model with sources of noise removed. In figure 6*a*, we show the time evolution of the distribution of *I*(*t*)/*A*(*t*) at the model best fit, along with the equivalent prediction from the homogeneous model. In figure 6*b*, we repeat this exercise for the total relative amount of antibody internalized, *I*(*t*). To understand uncertainty in these distributions, in figure 6*c*, we show the time evolution of the distribution of *I*(*t*)/*A*(*t*) alongside credible intervals formed by resampling parameters from the posterior distribution.

While our analysis revealed that several parameters are non-identifiable, or cannot be constrained to a relative precise interval, we are still able to produce relatively precise predictions of statistics such as the proportion of material internalized. Results in figure 6*c* show a discrepancy between predictions from the homogeneous and heterogeneous models. Aside from very early time, when the distribution of material internalized is relatively wide, the homogeneous model predictions lie within the lower tail of the predicted distribution. This is consistent with the discrepancy we observe in estimates of *p* between models: the heterogeneous model predicts that antibody disassociation and receptor recycling is rarer that what is predicted by the homogeneous model. This results in a smaller proportion of surface-bound antibody at late time.

Using the inferred joint distribution of *λ* and *β* we can build a picture of the proportion of receptors present on the cell surface at equilibrium (i.e. at the start of the experiment), *S*(0) (equation (2.4)). In figure 6*d*, we show the inferred distribution of *S*(0) at the model best fit, along with the uncertainty associated with the estimate and that predicted by the homogeneous model. While we have not precisely estimated this distribution, it is clear that, on average, a smaller number of receptors are present on the cell surface than not, in agreement with the prediction of the homogeneous model of 31%. We also see that the inferred distribution is highly variable; at the best fit, for example, non-zero density at zero suggests that some cells have a very small proportion of receptors on the surface, perhaps due to inhibition of recycling. In figure 6*e*, we show the inferred relationship between *S*(0) and *R* at the best fit. This result suggests that cells with a larger number of receptors—which may correlate to cells in latter stages of the cell cycle—have fewer surface-bound receptors.

## 3. Discussion

Heterogeneity is ubiquitous in cell processes such as the internalization of material, yet the phenomenon is poorly understood and often ignored. Paired with experimental protocols that probe these processes, flow cytometry is capable of generating vast quantities of single-cell snapshot data that capture cell-to-cell variability. Often, such data are summarized with point statistics that provide information about the transient behaviour to the detriment of acknowledging variability between otherwise homogeneous cells. In this study, we develop a mathematical model of internalization that captures dynamical behaviour, biological variability, and measurement noise of arbitrary magnitude. We apply our model to identify key sources of biological variability in the internalization of anti-TFR antibody by C1R B-cell lymphoblastoid cells.

While computationally costly, our distribution-matching ABC approach to inference carries several advantages over likelihood-based approaches; for example, those based on Bayesian hierarchical models or those that model cell properties as a finite mixture [39]. Firstly, ABC is robust to model error, incorporating uncertainty due to factors that are not explicitly modelled [52] by approximating the likelihood through an acceptance criterion that allows for an imperfect (i.e. *d*( · , · ) > 0) match between simulated and observed data. This might include the relatively small discrepancies we observe in figure 4 that highlight potential model-misspecification as well as error introduced experimentally, such as the precise measurement time and the time at which internalization is ceased.

Secondly, the distribution-matching approach allows the interpretation of pre-processed or summarized data, in contrast to typical techniques that require single-cell-level data. Automatic clustering algorithms [53–55] are an alternative to manual gating and provide an opportunity to analyse the parametric mixture distributions identified algorithmically, rather than relying on accurate classification of individual data points to perform analysis on the underlying data. Matching distributions rather than single-cell observations also carries a computational advantage, as, aside from initial data pre-processing, the approach is independent of the sample size.

Lastly, since ABC relies only on model simulations, our approach is agnostic to the complexity of the underlying measurement model and the signal-to-noise ratio. While the signal-to-noise ratio in our data is relatively high (demonstrated by the high correlation between BODIPY FL and FIP-Cy5 measurements in figure 1*d*), this is not always the case. In particular, flow cytometry measurements are often corrupted by autofluorescence and bleed-through from overlapping emission spectra. In our framework, both sources of extrinsic variability can be built into the probabilistic observation process that relates antibody concentration to flow cytometry measurements (equations (2.8)–(2.11)), or accounted for using pre-processing software where the compensated distributions are analysed rather than the underlying data.

**Figure 7.**
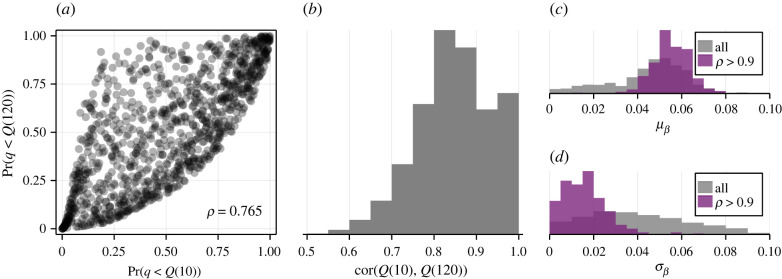
Dependence between the amount of material internalized at successive observation times remains uncertain. We demonstrate model predictions and uncertainty in the dependence between the noise-free quenched signal at time *t* = 10 and *t* = 120 min. (*a*) Quantile–quantile plot for a simulation with 1000 cells at the best fit. Correlation of *ρ* = 0.765 is calculated based on a fitted Gaussian copula. (*b*) Uncertainty in the inferred correlation based on resampled posterior samples. (*c*,*d*) Assuming strong dependence between observation times affects inferences. We show the posterior distribution for the recycling rate mean and standard deviation parameters, $\mu_{\beta }$ and $\sigma_{\beta }$, respectively, if we assume a correlation of at least 0.9.

Working with single-cell snapshot data collected using flow cytometry provides little to no information about the joint distribution of antibody concentration in individual cells between time points, potentially explaining why inferences relating to heterogeneity in dynamical parameters are relatively imprecise. Additional results in figure 7 illustrate the predicted dependence in internalized antibody concentration between early (10 min) and late (120 min) observation times, denoted by *Q*(10) and *Q*(120), respectively. An interpretation of model predictions with higher fitted correlations between *Q*(10) and *Q*(120) is that single-cell trajectories remain ordered: cells with a relatively lower proportion of antibody internalized at *t* = 10 min retain a relatively lower proportion at *t* = 120 min. Therefore, it is unclear from single-cell snapshot data (figure 7*b*) whether cell trajectories remain ordered or whether cells can ‘catch up’; that is, whether cells that are initially slow to internalize material end up with a large amount internalized at later time points. Intuitively, assuming that such a correlation is strong (i.e. trajectories remain ordered) strongly impacts inferences. Our results in figure 7*c*,*d* show that making such an assumption narrows uncertainty in the distribution of recycling rates to distributions where cells that do not recycle (i.e. *β*_*i*_ = 0) are rare. Single-cell trajectory data, collected through fluorescence microscopy [7], for example, could be applied in future to validate predictions relating to the joint distributions of fluorescence between time points, in addition to validating the inferred distributions for receptor count and the internalization and recycling rates.

Aside from stochastic variations between otherwise genetically identical cells—due to gene expression [56], for example—variability in internalization is at least partially driven by the cell cycle [57,58]. Therefore, we might expect lower internalization and recycling rates in cells preparing to undergo mitosis which, therefore, have a larger number of receptors. This is also suggested by results relating to the best fit in figure 5, which show that the internalization and recycling rates decrease with the number of receptors. A limitation of our model is that we cannot capture non-Gaussian dependence between the dynamical rate parameters without modelling subpopulations through a finite-mixture approach, which would significantly increase the dimensionality of the parameter space. For example, the dependence between *R* and *λ* may not be Gaussian, or even monotonic: internalization by cells in very late stages of the cell cycle might be inhibited, whereas in general, larger cells may internalize material more quickly [59]. Distribution-free approaches [39] might better capture the dependence structures in these cases. However, given that our model is already able to match the experimental data, adding complexity will exacerbate parameter non-identifiability. Therefore, further work should focus on experimental design [60]; by inhibiting recycling, pre-sorting cells to remove variability in *R* or working with single-cell trajectory data.

Our analysis demonstrates that inferences drawn using approaches that neglect heterogeneity can be misleading. In particular, the interpretation of predictions and parameter estimates from the homogeneous model are mathematically unclear. Generally, realizations of the homogeneous model do not represent the mean of realizations of the heterogeneous model, nor do they represent realizations where parameters in the heterogeneous model are first averaged [36]. While, in our case, parameters identified by the homogeneous model are contained within the distribution identified by the heterogeneous model, the homogeneous model produces biased predictions that are not representative of the entire population (figure 6). These findings highlight a need to co-develop mathematical tools that account for biological variability in analysis of single-cell data.

A better understanding of heterogeneity in internalization has important implications for drug delivery [5,19], in addition to our understanding of pathological processes, such as the internalization of viruses [61,62]. In this study, we develop a novel quantitative model that captures biological variability in internalization using arbitrarily noisy flow cytometry data. In contrast to conventional approaches, we can produce predictions that give insight into the variability in material internalized while accounting for inferential uncertainty. Applying mathematical models that capture biological variability allows practitioners to get the most out of the vast amounts of single-cell data generated by flow cytometry and other modern experimental tools.

## 4. Methods

### 4.1. Cell culture

C1R cells, a human B cell lymphoblast cell line, were cultured in Dulbecco’s modified Eagle medium (DMEM) supplemented with 10% FBS and 1% penicillin streptomycin, at 37°C in a humidified 5% CO_2_ atmosphere.

### 4.2. Dual-labelled fluorescent internalization probe

Purified monoclonal IgG1 anti-human transferrin receptor antibody (OKT9) [63] was purchased from WEHI Antibody Facility.

The antibody was labelled with two fluorescent dyes: BODIPY FL and FIP-Cy5. For this, anti-TFR antibody was incubated with BODIPY FL-NHS ester and incubated at 4°C overnight. BDP FL-labelled antibody was purified using a 7K MWCO Zeba spin desalting column (Thermo Scientific). The antibody was then functionalized with dibenzylcyclooctyne (DBCO)-NHS ester. Functionalized antibody was purified using a 7K MWCO Zeba spin desalting column (Thermo Scientific), and incubated with azide-FIP-Cy5 at 4°C overnight [64]. The dual-labelled antibody was purified using a 50K MWCO Amicon filter (Merck, Millipore), and the degree of labelling was measured by a NanoDrop UV–visible spectrophotometer.

### 4.3. Internalization assay

SHIP internalization assays were performed by incubating the cells with dual-labelled anti-TFR antibody in DMEM containing 0.1% FBS at 37°C for different time points. After incubation, cells were washed thrice with cold PBS and resuspended in propidium iodide with or without quencher (1 μM), as described previously [64]. Cells were analysed using a Stratedigm S1000EON flow cytometer and FlowJo 10.8.0.

## Data Availability

Code and data are available on GitHub at https://github.com/ap-browning/internalisation. The data are provided in electronic supplementary material [65].
